# Necrotizing Fasciitis in Northern Italy: Clinical Characteristics, Risk Factors, and Prognostic Value of the LRINEC Score—A Single-Center Retrospective Case Series

**DOI:** 10.3390/idr18030048

**Published:** 2026-05-18

**Authors:** Aurelia Sangani, Flavia Puci, Davide Tirro, Simona Villani, Camilla Torriani, Enrico Brunetti, Raffaele Bruno, Elisabetta Pagani

**Affiliations:** 1 Department of Clinical-Surgical, Diagnostic and Pediatric Sciences, University of Pavia, 27100 Pavia, Italy; aurelia.sangani01@universitadipavia.it (A.S.); flavia.puci01@universitadipavia.it (F.P.); davide.tirro@gmail.com (D.T.); enrico.brunetti@unipv.it (E.B.); raffaele.bruno@unipv.it (R.B.); 2 Infectious Diseases Unit I, IRCCS Policlinico San Matteo Foundation, 27100 Pavia, Italy; 3 Pediatric Clinic, IRCCS Policlinico San Matteo Foundation, 27100 Pavia, Italy; 4 Department of Public Health, Experimental and Forensic Medicine, University of Pavia, 27100 Pavia, Italy; simona.villani@unipv.it (S.V.); camilla.torriani01@universitadipavia.it (C.T.)

**Keywords:** necrotizing fasciitis, LRINEC score, Fournier gangrene, diabetes mellitus, Charlson Comorbidity Index, empirical antibiotic therapy, retrospective case series

## Abstract

Background: Necrotizing fasciitis (NF) is a rapidly progressive, life-threatening soft tissue infection characterized by fascial necrosis, with mortality rates of 20–30%. Despite its rarity, NF is increasingly encountered due to the rising prevalence of predisposing factors. Data from Southern European tertiary centers remain scarce. Methods: We retrospectively reviewed all patients ≥18 years with radiological and/or surgical diagnosis of NF managed at IRCCS Policlinico San Matteo, Pavia, Italy, between November 2018 and August 2023. Clinical, microbiological, and treatment data were extracted from electronic medical records. The Laboratory Risk Indicator for Necrotizing Fasciitis (LRINEC) score was calculated retrospectively. The Charlson Comorbidity Index was computed for each patient. Given the small sample size, we adopted a purely descriptive analytical approach without inferential testing. Results: Thirteen patients met inclusion criteria (median age 58 years, IQR 44.5–79.5; 69.2% male). The most common comorbidities were diabetes mellitus (6/13, 46.2%), renal failure (4/13, 30.8%), and chronic liver disease (4/13, 30.8%). The age-adjusted Charlson Index ranged from 0 to 11 (median 4). Lower limbs were the most frequently affected anatomic site (5/13, 38.5%), followed by the perineal/genital region (Fournier gangrene, 4/13, 30.8%). Type II (monomicrobial) NF predominated (9/13, 69.2%). Microbiological cultures were positive in 8/13 patients (61.5%): Gram-positive cocci were isolated in 5/8 (62.5%) and mixed aerobic/anaerobic flora in 3/8 (37.5%). Empirical antibiotic regimens included a piperacillin–tazobactam backbone in 6/12 (50.0%) patients and a meropenem-based combination in 5/12 (41.7%); 6/12 patients underwent targeted de-escalation after culture results. Two patients (15.4%) died in hospital, both with Fournier gangrene and Type I infection (mortality 2/4, 50.0% in Type I vs. 0/9 in Type II). The median length of stay was 26 days (IQR 17–28.5). All patients had LRINEC ≥6 at admission, with 9/13 (69.2%) classified as high risk (≥8). Conclusions: In this small retrospective Italian cohort, NF was most frequently associated with diabetes and high comorbidity burden. Type I (polymicrobial) infections, predominantly involving the perineal region, showed worse outcomes than Type II infections. The clinical experience accumulated during this study period subsequently informed the development of an institutional empirical antimicrobial protocol for skin and soft tissue infections at our hospital.

## 1. Introduction

Necrotizing fasciitis (NF) is a rapidly progressive infection of subcutaneous tissue and fascia, with mortality reported between 20% and 30% in most series and exceeding 50% when diagnosis or surgical intervention is delayed [1,2,3]. In Europe, the estimated incidence is 1.1–1.4 cases per 100,000 person-years; in Italy specifically, reported incidence ranges from 0.15 to 0.55 cases per 100,000 [4]. Despite recognized clinical importance, contemporary epidemiological and management data from Southern European tertiary centers are limited, with most published series originating from Northern Europe, North America, and Southeast Asia.

Diagnosis remains challenging because the early presentation is often indistinguishable from cellulitis or simple abscess [5,6]. Predisposing conditions include diabetes mellitus, peripheral vascular disease, chronic kidney disease, cirrhosis, malignancy, immunosuppression, and intravenous drug use [5,6]. Based on microbial etiology, NF is conventionally classified into four types [7]: Type I is polymicrobial, typically combining Gram-positive cocci and Gram-negative anaerobes (*Bacteroides fragilis*, *Prevotella* spp., *Fusobacterium* spp., *Peptostreptococcus* spp.), and most commonly affects diabetic and vasculopathic patients with abdominal, perineal, or trunk involvement; Type II is monomicrobial, most often caused by *Streptococcus pyogenes* (group A *Streptococcus*) or other beta-hemolytic streptococci such as *Streptococcus dysgalactiae*, and typically follows trauma or surgery to the extremities; *Staphylococcus aureus* (including methicillin-resistant strains) is an uncommon Type II pathogen; Type III is associated with marine-related Gram-negative organisms (notably *Vibrio* spp.); Type IV is fungal and occurs in immunocompromised hosts.

The Laboratory Risk Indicator for Necrotizing Fasciitis (LRINEC) score, developed by Wong et al. in 2004 [8], integrates six routine laboratory parameters (C-reactive protein, white blood cell count, hemoglobin, sodium, creatinine, and glucose) to support early discrimination of NF from other severe soft tissue infections. While useful as an adjunctive tool, its diagnostic sensitivity has been variable across validation studies (43–77%) [9,10], and recent evidence has shifted attention toward its potential prognostic role rather than purely diagnostic accuracy. Newer scoring systems such as SIARI (Site other than lower limb, Immunosuppression, Age, Renal impairment, and Inflammatory markers) [9] and the recently described NECROSIS score [11] have been proposed as complementary tools, though their applicability in retrospective cohorts is limited by data availability.

The primary objective of this study was to describe the epidemiological, clinical, microbiological, and therapeutic characteristics of NF cases managed at the IRCCS Policlinico San Matteo Hospital Foundation between 2018 and 2023. Secondary objectives were to describe the empirical and targeted antibiotic strategies adopted at our institution during the study period and to characterize patient outcomes by NF type.

## 2. Methods

### 2.1. Study Design and Setting

This is a retrospective, single-center, observational case series conducted at the IRCCS Policlinico San Matteo Hospital Foundation, a tertiary referral center in Pavia, Northern Italy. All adult patients presenting to the Emergency Department (ED) between November 2018 and August 2023 with clinically suspected NF were screened for inclusion. The study is reported in accordance with the Strengthening the Reporting of Observational Studies in Epidemiology (STROBE) guidelines [12], adapted for retrospective case series (Supplementary Table S1).

### 2.2. Ethics Statement

The study was approved by the Ethics Committee of the IRCCS Policlinico San Matteo (Protocol No. 0012532/26, approved on 25 February 2026). Given the retrospective design, the Committee granted a waiver of specific informed consent. Data were managed in accordance with the Declaration of Helsinki and Italian privacy legislation (Legislative Decree 196/2003, as amended by Legislative Decree 101/2018 implementing the EU GDPR).

### 2.3. Participants and Case Definition

Patients were identified through systematic review of ED records and hospital discharge databases using ICD-9-CM codes 728.86 (necrotizing fasciitis) and 608.83 (Fournier gangrene). Inclusion criteria were: (1) age ≥ 18 years; (2) presentation to the ED during the study period; and (3) confirmed NF diagnosis based on at least one of: (a) radiological evidence on computed tomography (CT) or magnetic resonance imaging (MRI) showing fascial thickening, fluid collections along fascial planes, or subcutaneous gas [13,14]; or (b) intraoperative findings of necrotic fascia, “dishwater-gray” fluid, friability of superficial fascia, or absence of pus, confirmed by the operating surgeon [7]. Exclusion criteria were: (1) age < 18 years; (2) absence of both radiological and surgical confirmation; and (3) incomplete medical records precluding data extraction.

**Figure 1:**
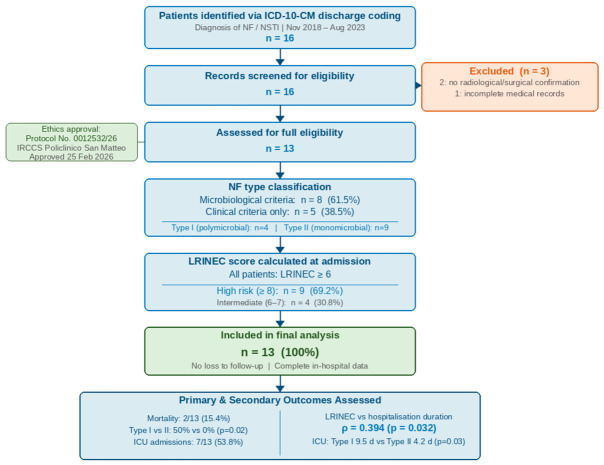
Patient selection flow diagram.

Sixteen patients with suspected NF were initially identified. Three were excluded: two lacked both radiological and surgical confirmation (one with multiple subcutaneous abscesses and one with subfascial abscess in the absence of fascial necrosis), and one had incomplete records. Thirteen patients were included in the final analysis (Figure 1).

### 2.4. Institutional Diagnostic and Management Pathway

At our institution, patients with clinically suspected NF presenting to the Emergency Department are evaluated jointly by the orthopedic surgeon and the Infectious Diseases consultant. Diagnostic imaging—computed tomography (CT) or magnetic resonance imaging (MRI)—is then obtained to confirm fascial involvement and define anatomic extension. The general surgeon is involved when wider debridement of the abdominal wall, trunk, or perineal region is required, and additional specialties (urology, otolaryngology, plastic surgery) are engaged according to the anatomic site. Throughout the study period (November 2018–August 2023), no formal institutional empirical antibiotic protocol for skin and soft tissue infections was in place; empirical regimens were therefore selected on a case-by-case basis by the treating physicians, integrating individual patient factors such as comorbidities, prior antibiotic exposure, suspected pathogen, allergy history, renal function, and known multidrug-resistant colonization. Microbiological sampling—including blood cultures and deep tissue cultures whenever feasible—was performed before antibiotic initiation when clinically possible, and de-escalation was performed once microbiological results became available, in accordance with antimicrobial stewardship principles. The clinical experience accumulated during the study period subsequently informed the development and implementation, in 2024, of a hospital-wide protocol of empirical antibiotic therapy (PDTA—Percorso Diagnostico Terapeutico Assistenziale) covering several infectious syndromes including skin and soft tissue infections [15]; this protocol post-dates the entire study period and therefore did not influence the management of the patients reported here. The clinical and microbiological diversity of our cohort is reflected in the heterogeneity of admitting departments (Infectious Diseases, General Surgery, Urology, Otolaryngology, Orthopedics and Traumatology), illustrating the importance of multidisciplinary collaboration in NF management.

### 2.5. Data Collection

Clinical data were extracted from electronic medical records by two investigators using a standardized data collection form. Variables collected included: age and sex; predisposing conditions (diabetes mellitus, peripheral vascular disease, obesity, intravenous drug use, HIV infection, renal failure, chronic liver disease, malignancy, recent surgery, pressure ulcers, transplant status, other immunosuppression); risk factors (penetrating and non-penetrating trauma, mucosal or skin breach); anatomic site of infection; admitting department; admission laboratory parameters; microbiological findings (from wound, blood, and tissue cultures); empirical and targeted antibiotic regimens; intensive care admission and duration; total length of hospital stay; and in-hospital outcome (survival or death). Discrepancies in extraction were resolved by consensus with a senior author.

### 2.6. NF Type Classification

Patients were classified as Type I (polymicrobial) or Type II (monomicrobial) based on microbiological culture results when available. For five patients (38.5%) without positive cultures—three with negative microbiological samples and two in whom cultures were not obtained before initiation of empirical antibiotics—classification was based on the combination of clinical presentation, anatomic site, and risk factor profile, in accordance with established classification systems [7,16]. Two investigators independently classified each case, with disagreements resolved by consensus with a third senior investigator. We acknowledge this approach as a methodological limitation and have reflected it in the discussion of study limitations.

### 2.7. LRINEC Score Calculation

The LRINEC score was calculated retrospectively using the first available laboratory values obtained at admission, according to the original scoring system [8]: C-reactive protein (mg/dL), total white blood cell count (×10^9^/L), hemoglobin (g/dL), sodium (mEq/L), creatinine (mg/dL), and glucose (mg/dL). Patients were stratified into established risk categories: low risk (≤5), moderate risk (6–7), and high risk (≥8). Because all patients in this series had confirmed NF, the LRINEC score could not be assessed for diagnostic discrimination (which would require a control group of patients with severe non-necrotizing soft tissue infection); rather, its distribution and association with clinical course are reported descriptively. The SIARI score was not retrospectively reconstructed because some of its required clinical components (notably documented immunosuppression status at the time of admission and exact baseline renal function trajectory) were not consistently recorded across all medical records.

### 2.8. Charlson Comorbidity Index

The age-adjusted Charlson Comorbidity Index was calculated retrospectively for each patient using documented comorbidities and age at admission, in accordance with the original weighting [17].

### 2.9. Statistical Analysis

In view of the small sample size (n = 13), all subgroups smaller than ten patients, and the consequent very limited statistical power, we adopted a purely descriptive analytical approach. Continuous variables are reported as median and interquartile range (IQR); categorical variables are expressed as absolute numbers and percentages. No inferential statistical testing (Fisher’s exact test, Mann–Whitney U test, or correlation coefficient with significance testing) was performed, in accordance with current methodological recommendations for small case series and the suggestions of the peer reviewers. All analyses were performed using R software (version 4.5.0, R Foundation for Statistical Computing, Vienna, Austria). Missing data were addressed via complete-case analysis; the handling of missing microbiological data is described in Section 2.6.

## 3. Results

### 3.1. Patient Characteristics

Thirteen patients met the final inclusion criteria. The median age was 58 years (IQR 44.5–79.5; range 36–87), with a male predominance (9/13, 69.2%). The most common comorbidities were diabetes mellitus (6/13, 46.2%), renal failure (4/13, 30.8%), and chronic liver disease (4/13, 30.8%). Three patients (23.1%) had peripheral vascular disease, three (23.1%) were obese, three (23.1%) had a history of intravenous drug use, two (15.4%) had pressure ulcers, and isolated cases (1/13, 7.7% each) had HIV infection, active malignancy, history of solid organ transplantation, or recent surgery. Only one patient had no recorded comorbidities. The age-adjusted Charlson Comorbidity Index ranged from 0 to 11 (median 4), reflecting a heterogeneous cohort with overall substantial comorbidity burden, particularly in elderly patients. Type II (monomicrobial) NF was the predominant type (9/13, 69.2%) (Table 1). Glycemic control could be assessed in 2 of the 6 diabetic patients with documented admission HbA1c values (13% and 11.1%, both indicating poor metabolic control); HbA1c was not consistently recorded for the remaining diabetic patients.

Patients were initially admitted to a heterogeneous range of departments: Infectious Diseases (4/13), General Surgery (3/13), Urology (2/13), Otolaryngology (1/13), Orthopedic and Traumatology (1/13), and other medical departments (2/13). This distribution reflects the heterogeneity of clinical presentations and the importance of cross-specialty awareness of NF.

### 3.2. Site of Infection

Lower limbs were the most frequent infection site (5/13, 38.5%), followed by the perineal/genital region (Fournier gangrene, 4/13, 30.8%), upper limbs (3/13, 23.1%), and the cervical region (1/13, 7.7%). No patient presented with thoracoabdominal or orbital involvement. The four patients with Fournier gangrene exhibited considerable phenotypic heterogeneity: one young man (36 years) presented without recorded comorbidities, with a minor skin breach as the only identifiable risk factor; an older patient (58 years) had a complex profile including HIV infection (CD4 count >1000 cells/μL with undetectable HIV-RNA on antiretroviral therapy), intravenous drug use, chronic viral hepatitis (HBV/HCV/HDV co-infection), and active malignancy; and the remaining two were elderly men (47 and 86 years) with multiple cardiovascular and metabolic comorbidities (Table 2).

### 3.3. Microbiological Findings

Microbiological isolation was achieved in 8/13 patients (61.5%). In the remaining five patients, cultures were either negative (n = 3) or not obtained before empirical antibiotic initiation (n = 2). Among patients with positive cultures, mixed aerobic-anaerobic flora was found in 3/8 (37.5%)—exclusively in Type I cases—while Gram-positive cocci predominated in 5/8 isolates (62.5%) and were associated with Type II infection (Table 3).

### 3.4. LRINEC Score Distribution

All thirteen patients had LRINEC scores ≥6 at admission. Nine patients (69.2%) were classified as high risk (LRINEC ≥8), while four patients (30.8%) fell in the moderate risk category (LRINEC 6–7). No patient had a low-risk score (Table 4). Because all patients had confirmed NF and no contemporaneous control group of patients with severe non-necrotizing soft tissue infection was available, the diagnostic discriminative ability of the LRINEC score could not be evaluated in this cohort. Patients with higher LRINEC scores tended to have longer hospitalizations, although the small sample size precludes formal inferential analysis. The median LRINEC score in survivors was 8 (range 6–10) and in non-survivors was 10 (both deceased patients had scores of 10).

### 3.5. Antimicrobial Therapy

Empirical antibiotic regimens were documented for 12/13 patients (one record was incomplete with respect to antibiotic data and is reported as such). During the study period—preceding the implementation of the institutional empirical antibiotic protocol [15]—regimens were chosen on a case-by-case basis according to the clinical judgment of the treating physicians. A piperacillin–tazobactam–based combination was used in 6/12 patients (50.0%), most frequently paired with vancomycin, daptomycin, or trimethoprim-sulfamethoxazole, with or without clindamycin. A meropenem-based combination was used in 5/12 patients (41.7%), typically with linezolid or vancomycin and clindamycin, in patients with prior multidrug-resistant colonization or recent broad-spectrum antibiotic exposure. One patient received a rifampicin–vancomycin combination as initial empirical therapy. Clindamycin was added to the empirical regimen in 6/12 patients (50.0%), reflecting the institutional practice of including an antitoxin agent in patients with suspected streptococcal etiology or severe systemic involvement [18]. No patient received empirical antifungal therapy or intravenous immunoglobulins (IVIG). Targeted de-escalation following microbiological results was performed in 6/12 patients (50.0%) (Table 5).

### 3.6. Clinical Outcomes

Two patients (15.4%) died during hospitalization. Both had Fournier gangrene with Type I (polymicrobial) infection. Mortality occurred in 2/4 patients with Type I NF (50.0%) and in 0/9 patients with Type II NF. The first deceased patient (47-year-old male) had peripheral vascular disease and obesity, with a length of stay of 52 days and death attributed to refractory septic shock. The second deceased patient (58-year-old male) had a complex baseline profile with HIV infection, intravenous drug use, chronic viral hepatitis (HBV/HCV/HDV), and active malignancy; he died of septic shock with multiorgan failure after 29 days of hospitalization. Among survivors, the median length of stay was 25 days (range 8–29). Type I infections were associated with longer median hospitalization than Type II (28 vs. 25 days), although the small sample size precludes formal comparison. All patients underwent surgical debridement within a short interval from admission (overall median 1 day, IQR 0–1, range 0–3); all four Type I patients were operated on the same day of admission, while Type II patients had a median interval of 1 day (IQR 1–1). The overall median length of stay was 26 days (IQR 17–28.5) (Table 6).

## 4. Discussion

In this single-center retrospective case series of 13 patients with confirmed NF managed over a five-year period in Northern Italy, three observations emerged: diabetes mellitus was the most common predisposing factor; Type II monomicrobial infections predominated and showed favorable outcomes; and Type I (polymicrobial) infections, all involving the perineal region or lower limbs, were associated with all in-hospital deaths. We discuss these findings against the published literature, with particular attention to the limitations of inferential interpretation in such a small sample.

### 4.1. Comorbidity Profile and Clinical Phenotype

The predominance of diabetes mellitus (46.2%) in our cohort is consistent with multiple international series identifying diabetes as the single most important predisposing condition for NF [19,20,21]. Diabetes contributes to NF risk through multiple pathogenic mechanisms, including microvascular ischemia, impaired neutrophil function, and reduced inflammatory response, often coexisting with peripheral vascular disease and chronic kidney disease [19]. The age-adjusted Charlson Comorbidity Index in our cohort showed considerable heterogeneity (median 4, range 0–11), illustrating the dual phenotype of NF: on one hand, elderly patients with cumulative metabolic and cardiovascular comorbidity, and on the other, younger patients (below 50 years) with specific and often severe individual risk factors such as intravenous drug use, chronic viral hepatitis, HIV infection, or simple skin breach in otherwise healthy individuals. This bimodal pattern is consistent with previous descriptions and underscores that NF should remain in the differential diagnosis of severe soft tissue infection across a wide range of clinical presentations.

### 4.2. Anatomic Distribution and Fournier Gangrene

The distribution of infection sites in our series, with lower limbs (38.5%) and perineal/genital region (30.8%) most commonly involved, is consistent with previous reports [7,16]. Both fatal cases involved Fournier gangrene with Type I infection. A recent systematic review reported mortality rates for Fournier gangrene ranging from 5.0% to 65.0%, confirming its position as a true urological emergency that occurs predominantly in men with diabetes or immunocompromised states [21,22]. We did not retrospectively calculate the Fournier Gangrene Severity Index (FGSI), as some of the required physiological parameters (notably arterial blood gas-derived bicarbonate and admission body temperature) were not consistently recorded at first ED contact for all patients. We acknowledge this as a limitation and consider FGSI a priority variable for prospective data collection.

### 4.3. Microbiological Findings and Empirical Antibiotic Choices

The successful microbiological isolation in only 8/13 patients (61.5%) reflects a recurrent challenge in NF series and is consistent with international reports. In our cohort, microbiological results aligned well with the conventional Type I/Type II dichotomy: mixed aerobic/anaerobic flora was found exclusively in Type I cases (3/3), while Gram-positive cocci accounted for all Type II isolates (5/5). This pattern reinforces the practical value of clinical and anatomic features for early classification when culture results are pending or unavailable. During the study period, empirical antibiotic regimens were chosen on a case-by-case basis by the treating physicians, in the absence of a formal institutional protocol for skin and soft tissue infections (which was developed and implemented at our institution only in 2024, after the end of the enrollment period [15]). The most frequently used schemes were a piperacillin–tazobactam backbone (50.0% of cases), generally paired with daptomycin or vancomycin to ensure activity against methicillin-resistant *Staphylococcus aureus* and serious streptococcal infections, and meropenem-based combinations (41.7%) reserved for patients with prior multidrug-resistant colonization or recent broad-spectrum antibiotic exposure. Clindamycin was added in 50.0% of regimens for its antitoxin effect, in line with current IDSA SSTI guidelines [18]. Targeted de-escalation following microbiological results was performed in 50.0% of cases, in line with antimicrobial stewardship principles. The favorable outcome of all Type II monomicrobial infections (mortality 0/9) suggests that this individualized, judgment-based approach provided adequate coverage in our cohort, and the experience accumulated during these years contributed to inform the subsequent development of an institutional empirical antibiotic protocol for skin and soft tissue infections at our hospital. We did not observe a clear association between specific empirical regimen and outcome in this small cohort, and given the very small sample size we refrain from any inferential claim. Linezolid was used in 3 of 12 patients (25.0%), in all cases combined with meropenem and clindamycin, and exclusively for cases with suspected polymicrobial infection in deeply contaminated sites (Type I). In our cohort, methicillin-resistant Staphylococcus aureus coverage in patients without prior colonization or specific risk factors was generally provided by vancomycin or daptomycin, with linezolid reserved for situations involving concurrent suspicion of vancomycin-resistant enterococci or anticipated difficult tissue penetration. The retrospective design and the small sample size do not allow inference on linezolid–outcome associations; we acknowledge that its toxin-suppressing properties and excellent tissue penetration could support a wider empirical role in selected NF cases, a question that the planned prospective registry may help clarify.

### 4.4. The LRINEC Score in a Contemporary Perspective

The LRINEC score has evolved from a primarily diagnostic tool to a more nuanced clinical decision aid. The original validation by Wong et al. [8] reported high diagnostic accuracy, but subsequent multicenter studies have reported significantly variable sensitivity (43–77%) [9,10], with a recent meta-analysis by Tarricone et al. reporting that the LRINEC score has high specificity (~83%) but limited sensitivity (~49%) for identifying necrotizing fasciitis, so that an LRINEC < 6 cannot safely rule out the diagnosis [23]. Recent work has emphasized its prognostic role: Hoesl et al. [2] demonstrated that higher initial LRINEC scores correlate with mortality risk, and that scores typically decline following adequate surgical debridement, with the largest decrement after the first intervention. Newer scoring systems such as the SIARI [9] and the recently described NECROSIS score have been proposed; the NECROSIS score, derived and validated by Kim et al. in a prospective EAST multicenter trial [11], has shown improved specificity and positive predictive value for NSTI identification in emergency general surgery cohorts. In our cohort, all patients had LRINEC ≥ 6 at admission, with both fatal cases scoring 10. Because all included patients had confirmed NF and no contemporaneous control group was available, the diagnostic discriminative ability of the score in our population could not be assessed; this represents an important limitation already raised by the peer reviewers, and a prospective design with parallel enrollment of patients with severe non-necrotizing soft tissue infections would be required to address it adequately.

### 4.5. Differential Outcomes by NF Type

The higher mortality observed in Type I compared with Type II infections (50.0% vs. 0.0%) is consistent with previous research suggesting that polymicrobial infections—particularly those involving the perineal region—carry a worse prognosis, possibly due to synergistic bacterial virulence factors and the anatomical complexity of perineal sepsis [7,16]. However, this pattern must be interpreted with great caution given the very small subgroup sizes (n = 4 and n = 9), which preclude any formal statistical inference. Our observation should be considered descriptive and hypothesis-generating, in line with the recommendations of the peer reviewers.

### 4.6. Strengths and Limitations

This study has several strengths. First, the case definition is rigorous, requiring radiological and/or surgical confirmation in all included patients. Second, data extraction was systematic and based on standardized criteria. Third, we provide detailed institutional information on the diagnostic pathway, empirical antibiotic regimens used in the pre-protocol era, and microbiological epidemiology in a Southern European tertiary center where such data are scarce.

Several important limitations must be acknowledged, many of which were appropriately raised by the peer reviewers. First, the very small sample size (n = 13) precludes inferential statistical analysis; we therefore present all results descriptively, in line with current methodological recommendations for small case series. Second, the retrospective design introduces inherent selection and information biases: patients with milder disease, atypical presentations, or those who died before reaching our institution may not have been captured. Third, as a tertiary referral center, our cohort likely reflects more severe and complex cases than community hospitals, limiting external generalizability. Fourth, the absence of a contemporaneous control group of patients with severe non-necrotizing soft tissue infection prevents evaluation of the diagnostic accuracy of the LRINEC score in our population; this should be addressed by future prospective studies with parallel enrollment. Fifth, the time of symptom onset before ED presentation—recognized as a key modifiable prognostic factor in NF—was not consistently retrievable from the available medical records, since it depended on patient-reported history of variable reliability. While the time from admission to first surgical debridement could be retrieved and is now reported (Section 3.6 and Table 1), the symptom-to-admission interval remains a major limitation of our retrospective dataset and one of the principal endpoints proposed for a planned prospective study. Sixth, the number of repeat debridements and the use of reconstructive surgery were not consistently documented and could not be analyzed. Seventh, the FGSI score was not retrospectively calculated due to incomplete physiological data at first ED contact. Eighth, microbiological data were missing or incomplete for 5/13 patients (38.5%), which may have influenced NF type classification despite our use of established clinical-anatomic criteria. Ninth, no long-term follow-up data—including 30- and 90-day mortality, functional status, wound healing, need for reconstructive surgery, or quality of life—were systematically available. Tenth, the SIARI score and NECROSIS score were not retrospectively calculated because some required components were not consistently recorded in our dataset.

### 4.7. Implications for Future Research

The limitations identified above directly inform the design of a planned prospective multicenter Italian NF registry, in which we propose: (a) standardized recording of all time intervals from symptom onset to first debridement; (b) systematic application of LRINEC, SIARI, NECROSIS, and FGSI scores at admission; (c) parallel enrollment of patients with severe non-necrotizing soft tissue infections as a comparator; (d) detailed documentation of empirical and targeted antimicrobial strategies, including measurement of adherence to the institutional empirical antibiotic protocol implemented in 2024 [15]; and (e) structured 30-day, 90-day, and 1-year follow-up. Such a registry could address the current paucity of Italian epidemiological data and provide a more robust evidence base for management algorithms.

## 5. Conclusions

In this small retrospective case series from a Northern Italian tertiary center, NF was most commonly associated with diabetes mellitus and a heterogeneous comorbidity profile. Type I (polymicrobial) infections, predominantly perineal, carried worse outcomes than Type II infections; both in-hospital deaths occurred in Type I Fournier gangrene cases. The LRINEC score was uniformly elevated (≥6) in all confirmed cases, supporting its potential clinical role although its diagnostic discriminative ability could not be assessed in this confirmed-NF cohort. Our description of the empirical antibiotic strategies adopted in the pre-protocol era—and the favorable outcome observed for all Type II monomicrobial infections—adds useful information from a Southern European setting where such data are limited. The clinical experience accumulated during the study period subsequently informed the development of an institutional empirical antibiotic protocol for skin and soft tissue infections at our hospital, implemented in 2024. These findings should be interpreted as descriptive and hypothesis-generating, given the small sample size and retrospective design. We propose a multicenter Italian NF registry with prospective design, standardized time-to-treatment recording, parallel non-necrotizing soft tissue infection comparator, and structured long-term follow-up to address the gaps identified by the present study. Both deceased patients had multiple severe predisposing conditions: one had HIV infection (well-controlled with high CD4 count and undetectable viral load), active malignancy, intravenous drug use, and chronic viral hepatitis (HBV/HCV/HDV co-infection); the other had peripheral vascular disease, obesity, recent surgery, and pressure ulcers. While neither patient met classical criteria for severe immunosuppression at the time of NF onset, the cumulative burden of host-defense impairment in both cases is consistent with the worse prognosis observed for Type I infection in our cohort.

## Figures and Tables

**Table 1:** Demographic data, comorbidities, and distribution by infection type (n = 13).

Variable	Overall (n = 13)	Type I (n = 4)	Type II (n = 9)
Median age, years (IQR)	58 (44.5–79.5)	56.5 (43.25–73.5)	59 (47–84)
Male, n (%)	9 (69.2%)	3 (75.0%)	6 (66.7%)
Diabetes mellitus, n (%)	6 (46.2%)	1 (25.0%)	5 (55.6%)
Peripheral vascular disease, n (%)	3 (23.1%)	1 (25.0%)	2 (22.2%)
Obesity, n (%)	3 (23.1%)	2 (50.0%)	1 (11.1%)
Intravenous drug use, n (%)	3 (23.1%)	1 (25.0%)	2 (22.2%)
HIV infection, n (%)	1 (7.7%)	1 (25.0%)	0 (0.0%)
Renal failure, n (%)	4 (30.8%)	1 (25.0%)	3 (33.3%)
Chronic liver disease, n (%)	4 (30.8%)	1 (25.0%)	3 (33.3%)
Malignancy, n (%)	1 (7.7%)	1 (25.0%)	0 (0.0%)
Recent surgery, n (%)	1 (7.7%)	1 (25.0%)	0 (0.0%)
Pressure ulcers, n (%)	2 (15.4%)	1 (25.0%)	1 (11.1%)
Solid organ transplant, n (%)	1 (7.7%)	0 (0.0%)	1 (11.1%)
Time from admission to first surgery, days, median (IQR)	1 (0–1)	0 (0–0)	1 (1–1)
Charlson Comorbidity Index, median (range)	4 (0–11)	1.5 (0–9)	5 (0–11)

Note: *p*-values removed; Charlson Comorbidity Index and admission-to-surgery interval added.

**Table 2:** Site of necrotizing fasciitis by infection type (n = 13).

Site	Overall (n = 13)	Type I (n = 4)	Type II (n = 9)
Upper limbs, n (%)	3 (23.1%)	0 (0.0%)	3 (33.3%)
Lower limbs, n (%)	5 (38.5%)	2 (50.0%)	3 (33.3%)
Perineal/genital, n (%)	4 (30.8%)	2 (50.0%)	2 (22.2%)
Neck/head, n (%)	1 (7.7%)	0 (0.0%)	1 (11.1%)

**Table 3:** Microbiological findings by infection type (n = 8 with positive cultures).

Pathogen Group	Overall (n = 8)	Type I (n = 3)	Type II (n = 5)
Mixed aerobic/anaerobic flora, n (%)	3 (37.5%)	3 (100.0%)	0 (0.0%)
Gram-positive cocci, n (%)	5 (62.5%)	0 (0.0%)	5 (100.0%)

Note: data corrected against source database; *p*-values removed.

**Table 4:** LRINEC score risk categories at admission (n = 13).

Risk Category	LRINEC Score	n (%)
Low risk	≤5	0 (0.0%)
Moderate risk	6–7	4 (30.8%)
High risk	≥8	9 (69.2%)

**Table 5:** Empirical antibiotic regimens used during the study period (n = 12 with available data).

Empirical Regimen	n (%)
Piperacillin–tazobactam + daptomycin (±clindamycin)	2 (16.7%)
Piperacillin–tazobactam + vancomycin (±clindamycin)	3 (25.0%)
Piperacillin–tazobactam + trimethoprim-sulfamethoxazole	1 (8.3%)
Piperacillin–tazobactam + clindamycin	1 (8.3%)
Meropenem + linezolid + clindamycin	3 (25.0%)
Meropenem + vancomycin (±clindamycin/metronidazole)	2 (16.7%)
Rifampicin + vancomycin	1 (8.3%)
Clindamycin included in any empirical combination	6 (50.0%)
Targeted de-escalation after culture results	6 (50.0%)

**Table 6:** Clinical outcomes by infection type (n = 13).

Outcome	Overall (n = 13)	Type I (n = 4)	Type II (n = 9)
Death, n (%)	2 (15.4%)	2 (50.0%)	0 (0.0%)
Recovery, n (%)	11 (84.6%)	2 (50.0%)	9 (100.0%)
Median length of stay, days (range)	26 (8–52)	28 (25–52)	25 (8–29)

Note: *p*-values removed.

## Data Availability

The de-identified datasets analyzed during this study are not publicly available due to patient privacy restrictions but may be made available from the corresponding author upon reasonable request and subject to Ethics Committee approval.

## Supplementary Materials

The following supporting information can be downloaded at: https://www.mdpi.com/article/10.3390/idr18030048/s1, Supplementary Table S1: Completed STROBE checklist for retrospective observational case series.

## Abbreviations

NF, Necrotizing fasciitis; LRINEC, Laboratory Risk Indicator for Necrotizing Fasciitis; DM, Diabetes mellitus; HIV, Human Immunodeficiency Virus; ED, Emergency Department; FG, Fournier gangrene; GAS, Group A Streptococcus; IQR, Interquartile range; IDU, Intravenous Drug Use; ICU, Intensive Care Unit; CT, Computed Tomography; MRI, Magnetic Resonance Imaging; STROBE, Strengthening the Reporting of Observational Studies in Epidemiology.
