# Supplementary material for: Necrotizing Fasciitis in Northern Italy: Clinical Characteristics, Risk Factors, and Prognostic Value of the LRINEC Score—A Single-Center Retrospective Case Series
Source: Infect Dis Rep. 2026 May 18;18(3):48. doi: 10.3390/idr18030048 (PMC13214898; doi:10.3390/idr18030048)
Supplement: Supplementary file 1 [file idr-18-00048-s001.zip › idr-4222170-supplementary.pdf]

Supplementary Table S1 — STROBE Statement Checklist

Section	Item	Recommendation / Compliance	Location
**Title and Abstract**	**1a**	Indicate study design in title or abstract. *Done.* "Single-Center Retrospective Case Series" stated in the title.	Title
	**1b**	Informative and balanced summary in the abstract. **[updated]** Now reports Charlson Index, corrected microbiological distribution, empirical antibiotic regimens, and length of stay; no inferential statistics.	Abstract
**Introduction**	**2**	Background and rationale. *Done.* European and Italian incidence; LRINEC rationale and limitations; NECROSIS score and the gap in Southern European data.	Section 1
	**3**	Specific objectives. **[updated]** Primary: clinical, microbiological, therapeutic characterization. Secondary: empirical antibiotic strategies and outcomes by NF type.	Section 1, last paragraph
**Methods**	**4**	Key elements of design. *Done.* Retrospective single-center case series, stated in first sentence of Methods.	Section 2.1
	**5**	Setting, locations, dates. *Done.* IRCCS Policlinico San Matteo, Pavia. November 2018 – August 2023.	Section 2.1
	**6a**	Eligibility, sources, selection. *Done.* Adults ≥18 with radiological/surgical NF confirmation. ICD-9-CM 728.86 and 608.83.	Section 2.3
	**6b**	Matching criteria. *N/A* — case series.	—
	**7**	Outcomes, exposures, predictors, confounders. **[updated]** LRINEC reported descriptively only — no diagnostic discrimination assessable without a non-NF comparator. Charlson Index calculated per Charlson 1987. NF type (I/II) classified by microbiology when available, otherwise by clinical-anatomic criteria.	Sections 2.6–2.8
	**8**	Sources of data and methods of assessment. *Done.* Standardized form, two extractors, consensus on disagreements.	Section 2.5
	**9**	Sources of bias. Two independent classifiers for the 5 culture-negative cases; consensus for disagreements. Other biases (single-center tertiary referral, retrospective design, no non-NF comparator) discussed in Section 4.6.	Section 2.6; Section 4.6
	**10**	Study size. *Done.* No formal calculation; all consecutive eligible cases over 5 years (rare disease).	Section 2.9
	**11**	Handling of quantitative variables. *Done.* Continuous: median (IQR). LRINEC categorised per Wong 2004 (≤5, 6–7, ≥8).	Section 2.9; Tables 1–6
	**12a**	Statistical methods. **[updated]** Descriptive analysis only. Inferential testing (Fisher, Mann-Whitney, Spearman) removed in this revision per reviewer recommendations. R v4.5.0.	Section 2.9

Section	Item	Recommendation / Compliance	Location
	**12b**	Subgroup and interaction analyses. **[updated]** Subgroup descriptions by NF type (n=4 vs n=9) reported as descriptive only, no inferential testing.	Sections 3.1–3.6; Section 4.5
	**12c**	Missing data. *Done.* Complete-case analysis. The 5 patients without positive cultures classified by clinical-anatomic criteria.	Sections 2.6, 2.9
	**12d**	Loss to follow-up. *N/A* — in-hospital follow-up complete for all 13 patients. Long-term follow-up unavailable (limitation 9).	Section 4.6
	**12e**	Sensitivity analyses. *None performed* — sample size precludes them.	Section 2.9
**Results**	**13a**	Numbers at each stage. *Done.* 16 identified, 3 excluded, 13 analysed.	Section 2.3; Figure 1
	**13b**	Reasons for non-participation. *Done.* 2 without confirmation, 1 incomplete records.	Section 2.3
	**13c**	Flow diagram. *Done.*	Figure 1
	**14a**	Participant characteristics. **[updated]** Table 1 includes age-adjusted Charlson Index (median 4, range 0–11) and distribution of admitting departments.	Section 3.1; Table 1
	**14b**	Missing data per variable. **[updated]** Microbiology missing 5/13; antibiotic regimen missing 1/13; admission-to-surgery interval missing for 1/13; symptom-to-admission interval, repeat debridements, FGSI parameters and long-term follow-up not consistently recorded.	Sections 2.6, 3.5; Section 4.6
	**14c**	Follow-up time. *N/A* — in-hospital case series.	—
	**15**	Outcome events. **[updated]** Mortality 2/13 (both Type I Fournier). LRINEC distribution. Antibiotic regimens (Table 5); LOS (Table 6).	Sections 3.4–3.6; Tables 4–6
	**16a**	Effect estimates and precision. **[updated]** Descriptive only — medians with IQR/range, percentages. No p-values, no adjusted estimates.	Sections 3.1–3.6; Tables 1–6
	**16b**	Category boundaries when continuous variables were categorised. *Done.* LRINEC tiers per Wong 2004.	Section 2.7; Table 4
	**16c**	Translation of relative risk into absolute risk. *N/A* — descriptive case series.	—
	**17**	Other analyses. **[updated]** Spearman correlation (LRINEC vs LOS) removed in this revision. No interactions or sensitivity analyses.	Sections 3.1–3.6
**Discussion**	**18**	Key results. **[updated]** Section 4 opens with three observations (diabetes predominance; Type II favorable outcomes; Type I deaths in Fournier). Discussion organized in seven thematic subsections.	Section 4 (intro); 4.1–4.7

Section	Item	Recommendation / Compliance	Location
	**19**	Limitations. **[updated]** Section 4.6 lists ten limitations: small sample, retrospective design, tertiary-center bias, no non-NF comparator, missing symptom-to-admission interval, repeat debridements, FGSI not calculable, missing microbiology in 5/13, no long-term follow-up, SIARI/NECROSIS not calculable.	Section 4.6
	**20**	Overall interpretation. **[updated]** Findings framed as descriptive and hypothesis-generating; contextualized against Tarricone 2022 meta-analysis and Kim 2024 NECROSIS validation.	Sections 4.1–4.5; Section 5
	**21**	Generalisability. **[updated]** Tertiary IRCCS center in Northern Italy; results not directly transferable to community settings. Institutional PTDA implemented in 2024 (post-study) opens prospective evaluation paths.	Section 4.6 (point 3); 4.7
**Other Information**	**22**	Funding. *Done.* No external funding.	Funding section
**Additional reporting items added in this revision (not standard STROBE)**			
**Institutional pathway**	—	**[new]** Joint orthopedic and Infectious Diseases evaluation, then CT/MRI, then general surgeon if needed. No formal SSTI antibiotic protocol during the study period; the institutional PTDA was implemented in 2024, after enrolment ended.	Section 2.4
**Comorbidity Index**	—	**[new]** Charlson 1987 weighting; reported in Table 1 and discussed in Section 4.1.	Section 2.8
**Antimicrobial therapy**	—	**[new]** Empirical regimens for 12/13 patients in Table 5: Pip-Tazo–based 50.0%, meropenem-based 41.7%, clindamycin in half. Targeted de-escalation in 50.0%.	Section 3.5; Table 5
**Future research**	—	**[new]** Multicenter Italian NF registry proposed: time-to-treatment, all NF scores, non-NF comparator, PTDA adherence, structured 30/90/365-day follow-up.	Section 4.7
**AI use declaration**	—	**[new]** No generative AI used in writing.	Declaration on AI Use

Items marked **[updated]** were revised in this submission to reflect changes from the original manuscript; **[new]** indicates content added in this revision. These red markers serve a semantic function only (signalling revision status).

Reference: von Elm E, Altman DG, Egger M, et al. The Strengthening the Reporting of Observational Studies in Epidemiology (STROBE) statement: guidelines for reporting observational studies. Lancet. 2007;370(9596):1453–1457.

EQUATOR Network: https://www.equator-network.org/reporting-guidelines/strobe/ (accessed on 1March 2026)
